# Association of -460C/T and +405 G/C polymorphisms of vascular endothelial growth factor gene and susceptibility to ovarian hyperstimulation syndrome

**Published:** 2017-02

**Authors:** Nasrin Ghasemi, Razieh Dehghani Firouzabadi, Shahnaz Ahmadi

**Affiliations:** 1 *Recurrent Abortion Research Centre, Reproductive Sciences Institute, Shahid Sadoughi University of Medical Sciences, Yazd, Iran. *; 2 *Research and Clinical Center for Infertility, Shahid Sadoughi University of Medical Sciences, Yazd, Iran.*; 3 *Iran University of Medical Sciences, Tehran, Iran.*

**Keywords:** Ovarian hyperstimulation syndrome, Vascular endothelial growth factor, Gene polymorphism

## Abstract

**Background:**

Ovarian hyperstimulation syndrome (OHSS) is one of the most important complications of assisted reproduction treatment. Many substances are involved in the regulation of the vascular permeability, which have been concerned to cause OHSS. Vascular endothelial growth factor (VEGF) has emerged as one of the main angiogenic factors, which could be responsible for increased vascular permeability.

**Objective:**

In this study the association of vascular endothelial growth factor -460C/T and +405 G/C polymorphisms and susceptibility to ovarian hyperstimulation syndrome was evaluated.

**Materials and Methods:**

In this cross sectional study, VEGF gene polymorphisms were amplified by Polymerase chain reaction- Restriction Fragment Length Polymorphism in 75 women with established OHSS (case group) and 85 normoresponder (control group) which received conventional ovarian stimulation regimen.

**Results:**

There was no significant difference in the frequency of -460 C/T polymorphism between cases and controls (p=0.85). The frequency of +405 G/C polymorphism was significantly higher in the OHSS women (p=0.03, OR=2.44; 95% CI=1.23-4.82).

**Conclusion:**

In women who developed OHSS, *VEGF* gene polymorphism +405 could be effective. Two of the polymorphisms -460 C/T and +405 G/C were reported to be associated with increased VEGF basal promoter activity. However, only +405 G/C gene polymorphisms were more frequent in cases than controls.

## Introduction

Ovarian hyperstimulation syndrome (OHSS) is one of the most important complications of assisted reproduction treatment (ART). There is no clinical relevance for mild OHSS, which could progress to severe type without treatment. Severe OHSS characterized by massive ovarian enlargement ascites, pleural effusion, oliguria, hemoconcentration, and thromboembolic phenomena, and it is a life-threatening complication (1, 2). The incidence of all forms of OHSS is highly variable in the range of 1-23%, and up to 5% of all treatment cycles are severe form (3). The pathophysiology of OHSS remains uncertain. Many substances involved in the regulation of the vascular permeability, which has been concerned to cause OHSS. The mechanism of OHSS has currently been explained on the basis of the sudden increase in capillary permeability, which is leading to rapid and massive fluid and electrolyte shift from the intravascular compartment to third spaces (4). Vascular endothelial growth factor (VEGF) has emerged as the main angiogenic factor, which is responsible for increased vascular permeability. It leads to the extravasation of protein-rich fluid and subsequently the full appearance of OHSS. Vasoactive properties and increased ovarian expression during the development of OHSS suggest that VEGF plays a major role in the development of this syndrome. Serum VEGF levels increase after Human chorionic gonadotropin (hCG) administration in superovulated women, which could be a risk of development OHSS (5-7). In women who developed OHSS, VEGF was released into the follicular fluid in response to hCG, which increased capillary permeability (8). VEGF is a member of a family of heparin-binding proteins that are directly on the endothelial cells to induce proliferation and angiogenesis (9).

Some polymorphisms of *VEGF* gene are up the regulator and some are down regulator. Polymorphisms -460 C/T and +405 G/C are up the regulator and were associated with increased *VEGF* basal promoter activity and enable to increase permeability (10-12). Polymorphisms in the *VEGF* gene could be associated with the occurrence of OHSS (13). Increased risk of OHSS was reported in patients with *VEGF* +405 C/C genotype (14).

It is logical to examine the relationship between the *VEGF* polymorphism and OHSS. This project was conducted as a cross-sectional study to evaluate the potential association between OHSS and *VEGF* -460 C/T and +405 G/C polymorphism.

## Materials and methods

A total of 160 infertile women aged 20-35 yr who referred to Research and Clinical Center for Infertility, Yazd, Iran for assisted reproductive techniques (ART) were enrolled in this case-control study. Women had body mass index (BMI) more than 30 kg/m^2^, and history of polycystic ovary syndrome was excluded. All participants were received conventional ovarian stimulation regimen in their ART cycle. They desensitized with buserelin (Suprefact, Aventis, Frankfurt, Germany) 500 µg subcutaneously (S.C.) in the 21 day of menstrual cycle, until the baseline evaluation, which takes place in the first few days of menstruation. If baseline levels of estradiol (<50 pg/ml ) had been achieved, then the dose of buserelin was reduced to 250 µg and ovarian stimulation was commenced with 150-225 IU recombinant Follicle-stimulating hormone (Gonal F, Serono, Aubnne, Switzerland) S.C for ART treatment. 

Our participants divided into two group according to high does OHSS defined as the presence of equal or more than 15 follicles with a mean diameter of equal or more than 12 mm per each ovary at the end of the follicular phase of controlled ovarian hyperstimulation, or E_2_ levels on the day of hCG administration more than 3000 pg/ml or 15 oocytes retrieved, accompanied by ascites in the embryo transfer’s day or until 14 days after that. 

**Laboratory evaluation**

From each participants, 5 ml peripheral blood sample in ethylenediaminetetraacetic acid was taken. DNA was extracted by salting-out method (15). *VEGF* gene was amplified by polymerase chain reaction (PCR), using 50 ng genomic DNA, 1×Taq polymerase, buffer (1.5 mM Mgcl_2_, Cinagen, Tehran, Iran) and 2-6 pmol of each primer (Cinagen, Tehran, Iran). The primers used for -460 gene polymorphism were forward 5'-TGTGCGTGTGGGGTTGAGCG-3' and reverse 5'-TACGTGCGGACAGGGC CTGA-3' (Cinagen, Tehran, Iran), and the primers used for +405 gene polymorphism were forward 5'-ATTTATTTTTGCTTGCC ATT-3' and reverse 5'- GTCTGTCTGTCTG TCCGTCA-3' (Cinagen, Tehran, Iran). PCR amplification was performed in a programmable thermal cycler (gradient PCR termocycler, Ependorf, Germany).

The cycling conditions for the *VEGF* gene -460 polymorphism were set as follows; 1 cycle at 94^o^C for 7 min, 35 cycles at 94^o^C for 30 sec, 55.5^o^C for 45 sec, and 72^o^C for 45 sec, and 1 final cycle for an extension at 72^o^C for 5 min. The 175-bp PCR product was mixed with 2 units of BstUI (Fermentaz, Life Science, U.K.) and the reaction buffer according to the manufacture’s instruction. The restriction site was located at -460 with a C polymorphism. The C form of PCR products should be digested, and the T form could not. 

Two fragments measuring 155 and 20 bp were present if the product was digested (C form). The reaction was incubated for 3 hours at 37^o^C followed by 20 min in 65^o^C for enzyme inactivation. Then, 10 µL of the product was loaded onto a 3% agarose gel containing ethidium bromide for electrophoresis. The genotype was categorized as C/C (digested), T/T (undigested), and C/T. 

The cycling conditions for the *VEGF* gene +405 polymorphism were set as follows; 1 cycle at 94^o^C for 5 min, 35 cycles at 94^o^C for 1 min, 53^o^C for 1 min, and 72^o^C for 1 min, and 1 final cycle for an extension at 72^o^C for 5 min. The 304 bp PCR product was mixed with 2 units of BsmFI (Fermentaz, Life Science, U.K.) and the reaction buffer according to the manufacture’s instruction. The restriction site was located at +405 with a G polymorphism. 

The G-form PCR products should be digested, and the C form could not. Two fragments measuring 193 and 111 bp were present if the product was digestible (G form). Then the enzyme inactivation and electrophoresis were done the same as -405 gene polymorphism. The polymorphism was categorized as G/G (digested), C/C (undigested), and G/C. 

**Ethical consideration**

This project was approved by the Ethics Committee of Research and Clinical Center for Infertility, Yazd, Iran, and all patients were required to sign a written informed consent a participating to this study.

**Statistical analysis**

Statistical Package for the Social Sciences (SPSS, version 15.0 for windows ; SPSS Inc., Chicago. IL) was used for data analysis. Genotype, allele frequencies were compared using Chi-square test. Statistical significance was taken as p<0.05. Odd ratios (ORs) were calculated with 95% confidence interval (CI).

## Results

A total of 160 infertile women were scheduled in this study (75 women with established OHSS as the case group and 85 normoresponder as controls). The mean±SD of the age in OHSS group was 27.4±2.7 yr and, 26.8±1.68 yr in controls. Mean±SD BMI was 24.5±1.9 kg/m2 in OHSS group and 24.2±1.5 kg/m^2^ in the control group. There were no differences in age and BMI between both groups. 

The genotype and allele frequency of the *VEGF* -460 polymorphism in the control group and OHSS group are shown in table I. There was no statistically significant difference in the *VEGF* -460 C/T polymorphism between the two groups in terms of genotype (p=0.85), and allele frequency (p=0.75). Table II lists the distribution of the genotype and allele frequency of the +405 G/C polymorphism in two groups. There was the statistically significant difference in the genotype frequency in this gene polymorphism (p=0.03). The frequency of the G/G genotype was significantly higher in the OHSS group (OR=2.44; 95% CI=1.23-4.82).

**Table I:** *VEGF* -460 genotypes and alleles frequency in the case and control groups

	**Genotypes**				**Alleles**		
	**C/C**	**C/T**	**T/T**	**Sum**	**C**	**T**	**Sum**
Cases (OHSS group)	57 (76%)	12 (16%)	6 (8%)	75 (100%)	126 (84%)	24 (16%)	150 (100%)
Controls (Normoresponder group)	65 (76.5%)	15 (17.6%)	5 (5.9%)	85 (100%)	145 (85.3%)	25 (14.7%)	170 (100%)

Chi-square test, (p= 0.85)

All data presented as n(%)

**Table II:** *VEGF* +405 genotypes and alleles frequency in the cases and controls groups

	**Genotypes**				**Alleles**		
	**G/G**	**G/C**	**C/C**	**Sum**	**G**	**C**	**Sum**
Cases (OHSS group)	15 (20%)	57 (76%)	3 (4%)	75 (100%)	87 (58%)	63 (42%)	150 (100%)
Controls (Normoresponder group)	30 (35%)	48 (56.5%)	7 (8.2%)	85 (100%)	108 (63.5%)	62 (36.5%)	170 (100%)

Chi-square test (p= 0.03) (OR=2.44; 95% CI=1.23-4.82)

All data presented as n (%)

## Discussion

This study evaluated the association between *VEGF* -460 C/T and +405 G/C polymorphism with OHSS. There are few studies in the world which try to find a relationship between *VEGF* gene polymorphism and OHSS (4-7). Two of the polymorphisms -460 C/T and +405 G/C were reported to be associated with increased *VEGF* basal promoter activity. This can be observed in proliferative diabetic retinopathy, acute respiratory distress syndrome, and endometriosis (10-12). 

-460 C/T and +405 G/C gene polymorphisms enable increase permeability and the mechanism of OHSS is increased capillary permeability, therefore, OHSS could be reduced using VEGF antagonist (16). Nouri *and colloquies* confirmed that *VEGF* gene polymorphism is related with the happening of OHSS (13). Even patients with *VEGF* +405 CC genotype have increased the risk of OHSS (14). 

However, Shadiran *et al*, and Ghazizadeh *et al* showed the relation of *VEGF* gene polymorphism with varicose vein and metabolic syndrome. Slatery *et al*, Scartozzi *et al,* and Shibuya *et al* confirmed that *VEGF* receptors gene polymorphism could be associated with cancer, storke, and systemic lupus erythematosus (17-19). In addition, Honarvar *et al* and Stu *et al* found the relation between this polymorphism with recurrent pregnancy loss (20, 21). 

It was shown that in women who develop OHSS, VEGF is overexpressed. It produces by lutein cells and induces increased permeability (22-25). The VEGF system is composed of one agonist part, two transmembrane, and one antagonist soluble receptors (26). The agonist part stimulates both angiogenesis and vasculogenesis. VEGF can induce all the process which are typical symptoms of OHSS. A major effect is the fenestration of vessels increasing vascular permeability (27). 

The granulose and endothelial cells may be involved in the higher production and release of VEGF in women treated with gonadotropins and develop OHSS. Analyzing the clinical course of OHSS, it becomes clear that a risk exists only after ovulation and during corpus leuteum formation (28). The patients complain of discomfort before ovulation due to enlarged cystic ovaries. 

The present study showed that there was a significantly higher rate of +405 G/C genotype in OHSS patients (OR=2.44). While it was unable to demonstrate a significant correlation between -460 gene polymorphism in OHSS group. It was demonstrated that low VEGF receptor (VEGFR) concentration coincided with high ovarian response (25). *VEGF* +405 polymorphism could be a target for VEGFR which appears to be mainly involved in regulating vascular permeability, angiogenesis, and vasculogenesis.

## Conclusion

In conclusion, *VEGF* +405 gene polymorphism is a risk factor for OHSS. Study of other polymorphism of *VEGF* gene could improve this problem. This information could be applied for diagnosis of the high-risk individuals for OHSS. 
